# Recruiting Women for a Study on Perceived Risk of Cancer: Influence of Survey Topic Salience and Early Versus Late Response

**DOI:** 10.5888/pcd10.120293

**Published:** 2013-05-09

**Authors:** Steven Leadbetter, Nikki A. Hawkins, Lawrence E. Scholl, Frances A. McCarty, Juan L. Rodriguez, Naomi Freedner-Maguire, Sharon Hensley Alford, Cecelia A. Bellcross, Lucy A. Peipins

**Affiliations:** Author Affiliations: Nikki A. Hawkins, Juan L. Rodriguez, Lucy A. Peipins, Centers for Disease Control and Prevention, Atlanta, Georgia; Lawrence E. Scholl, Naomi Freedner-Maguire, ICF International, Fairfax, Virginia; Frances A. McCarty, Cecelia A. Bellcross, Emory University, Atlanta, Georgia; Sharon Hensley Alford, Henry Ford Health System, Department of Public Health Science, Detroit, Michigan.

## Abstract

**Introduction:**

Understanding the characteristics of early and late survey responders has implications for recruitment efforts and for informing potential response bias. The main objective of this analysis was to examine survey responder status (ie, early vs late response) by sociodemographic characteristics and by salience of study variables among respondents.

**Methods:**

We analyzed data from a survey on family cancer history and perceived cancer risk among women at a large managed health-care organization. For baseline and 12-month follow-up surveys, we defined early versus late responder status according to the 95th percentile of the number of days it took to obtain completed interviews.

**Results:**

We found no significant associations between responder status and sociodemographic characteristics at baseline or follow-up. At baseline, early responders were significantly more likely than late responders to have a personal history of breast cancer (5.2% vs 3.4%, *P* = .04) and to have been referred for genetic counseling (4.6% vs 2.0%, *P* = .004). The association between personal history of breast cancer and responder status persisted at follow-up; only 3.5% of late responders at baseline were also late responders at follow-up. Follow-up survey nonresponse rates did not vary by baseline responder status.

**Conclusion:**

Survey topic salience is associated with early response and is important for recruitment. However, once recruited, late responders do not remain late responders at follow-up, suggesting that extra efforts made to recruit late responders are worthwhile. Health-related agencies that conduct surveys should consider survey salience in survey administration and recruitment strategies.

## Introduction

Nonresponse bias in survey research can affect the validity of interpretation and generalizability of results (1). To achieve desirable response rates, researchers have learned to design and implement studies that maximize perceived benefits, minimize costs, and promote trust among respondents — an approach that posits survey response as the outcome of a social exchange where costs and benefits of an interaction are weighed by participants (2). Mechanisms underlying response include elements such as questionnaire design and content, participation incentives, and thank-you letters. Several studies have reported demographic differences by early versus late response status (3–6), whereas others have shown little or no differences (7,8). Inconsistencies in response-related demographic characteristics are not entirely surprising because target populations, survey designs, types of administration, and survey topic areas vary across studies.

One key predictor in survey nonresponse is the salience of the research topic to potential participants (9,10). Groves and colleagues (11) proposed a conceptual framework for survey participation that incorporates topic of interest or salience, sponsoring organization, and incentives as factors that leverage cooperation. Critical in this leveraging process is a study introduction that invokes relevance based on respondents’ personal characteristics or experiences.

The objective of this analysis was to examine survey responder status (early vs late) by sociodemographic characteristics and by salience of study variables among respondents. Because late response in an initial survey might indicate reluctance to respond to follow-up surveys, we also examined whether late responders at baseline were more likely to be late responders or nonresponders at follow-up. Understanding the characteristics of early and late responders has implications for recruitment efforts and potential response bias.

## Methods

### Study setting and design overview

As part of a larger study by the Centers for Disease Control and Prevention (CDC) of perception of ovarian cancer risk and cancer screening, we conducted a survey of women from the Henry Ford Health System (HFHS), a large, integrated health system serving the Detroit metropolitan area. Using a survey design consisting of baseline and 12-month follow-up telephone interviews, we surveyed women on their personal history of cancer, perceived risk of ovarian cancer, cancer screening history, genetic counseling referrals in their families, and demographics. Participants provided their family history of breast and ovarian cancer in first- and second-degree relatives. In the follow-up survey, we assessed whether intent to undergo screening at baseline was carried out and inquired about any changes in family history of cancer and perceived risk since the baseline interview. We obtained study approvals from the institutional review boards of CDC and HFHS. Because we asked respondents potentially sensitive questions, we obtained a 301(d) Certificate of Confidentiality of the Public Health Service Act to provide additional confidentiality assurances. We obtained informed consent from all respondents before conducting interviews.

### Recruitment

An introductory letter sent to potential participants before recruitment stated that HFHS was working with CDC to learn more about what women know and think about cancer, including their experiences with cancer in their family, worries they might have about cancer, and perceptions of their own risk of cancer. The letter explained that the study results might help researchers understand how women make decisions about their health, who is screened for cancer, and why certain women undergo screening. The letter indicated that the research findings could provide information to doctors on better ways of talking about cancer screening with their patients. Telephone interviews commenced approximately 1 week after the introductory letters were distributed.

### Eligibility screening and baseline survey implementation

The sampling frame included women aged 30 or older in the HFHS master patient index who did not have a known history of ovarian cancer (Figure). These women were randomly assigned to 1 of 6 smaller list frames in separate recruitment waves. We used a computer-assisted telephone interviewing (CATI) system to contact women and give them information about the research study, similar to the information provided in the introductory letter. We administered a brief eligibility screener, asking about personal breast or ovarian cancer history, bilateral oophorectomy, and the number of breast and ovarian cancer cases among first- and second-degree relatives. Women reporting a history of ovarian cancer or bilateral oophorectomy were not eligible. Responses to questions about personal and family history of cancer enabled immediate classification of women into preliminary high, elevated, and average risk groups for stratified random sampling purposes, where the CATI system randomly selected potential respondents for full baseline interview participation. After consent, baseline survey interviews took approximately 35 minutes to administer. Baseline interviewing began January 16, 2008, and concluded December 10, 2008.

**Figure:**
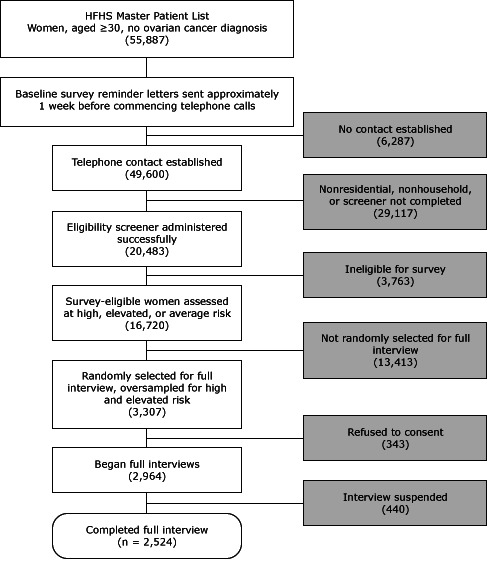
Baseline survey administration: screening, sampling, and obtaining full interviews. A computer-assisted telephone interview system randomly selected survey-eligible women for full interview in real time; we consented participants and conducted full interviews immediately after screening. All such activities generally occurred during 1 phone call. Abbreviation: HFHS, Henry Ford Health System.

Discounting for refusals and unsuccessful contact attempts, we screened 20,483 of the 55,887 women on the HFHS master list frame (36.7% screening rate), of whom 16,720 (81.6%) were deemed eligible for the study. Randomly selecting and inviting 3,307 eligible women to participate, we successfully consented and interviewed 2,524 women (76.3% response rate). Participants completing the baseline survey received a $15 gift card and were invited to participate in the follow-up survey, conducted 1 year later.

### Follow-up survey implementation

Baseline participants who expressed interest in completing a follow-up survey were sent a $10 cash incentive enclosed in a follow-up survey reminder letter. Follow-up survey interviews on average took less than 10 minutes to administer and were conducted February 18, 2009, through February 3, 2010. Among the 2,524 baseline survey respondents, 2,474 women expressed interest in participating in the follow-up survey, 1,910 of whom completed the follow-up (75.7% response rate).

### Objective risk classification

We based our final ovarian cancer risk classification on the US Preventive Services Task Force (USPSTF) criteria for BRCA1/2 mutation genetic counseling (12). To classify women at high risk, we used scenarios based on counts of first- or second-degree relatives from the same side of the family (maternal or paternal) who were diagnosed with breast or ovarian cancer. An elevated risk category included women whose family histories were not diagnostic of hereditary cancer but potentially indicative of a higher risk for breast and ovarian cancer. A personal history of breast cancer was indicative of either high or elevated risk, depending on age at diagnosis and heritage (13–16). Any first-degree relative or second-degree relative diagnosed with breast cancer or ovarian cancer for women of Ashkenazi-Jewish heritage indicated increased (high or elevated) risk. We classified women as average risk if they had very limited or no family history of breast or ovarian cancer (Box).

**Box. High and Elevated^a ^Objective Risk Group Classification Scenarios,^b^ Henry Ford Health System, Non-Jewish^c^ Women, Aged 30 or Older, 2008**

**Table:** 

**Objective Risk Classification Scenarios**
**High risk**
2 First-degree relatives with breast cancer, at least 1 of whom had been diagnosed at age ≤50
Combination of 3 or more first-degree relatives or second-degree relatives with breast cancer
Combination of 2 breast cancers and ovarian cancers among first-degree and second-degree relatives
1 First-degree relative with bilateral breast cancer
1 First-degree relative or 1 second-degree relative with both breast cancer and ovarian cancer
Breast cancer history: 1 first-degree male relative or 1 second-degree male relative
Personal history of breast cancer, diagnosed at age ≤50
2 Ovarian cancers among first-degree relatives or second-degree relatives (2 first degree, 2 second degree, or 1 first degree and 1 second degree)
1 First-degree relative and 1 second-degree relative with breast cancer, both diagnosed at age ≤50
2 Second-degree relatives on the paternal side with breast cancer, both diagnosed at age ≤50
**Elevated risk**
1 First-degree relative with ovarian cancer
2 Second-degree relatives with breast cancer (except 2 paternal, both diagnosed at age ≤50)
Personal history of breast cancer, diagnosed at age >50
2 First-degree relatives with breast cancer, both diagnosed at age >50
1 First-degree relative and 1 second-degree relative with breast cancer, at least 1 relative diagnosed at age >50
1 First-degree relative with breast cancer, diagnosed at age ≤50

**^a ^** All women classified at average objective risk had very limited or no family history of breast or ovarian cancer.

**^b ^** Breast cancer or ovarian cancer diagnosed at any age if an age group is not specified. All family history scenarios refer to female relatives except for the high-risk scenario of breast cancer among male relatives.

**^c ^** Ashkenazi-Jewish women with a personal history of breast cancer at any age of diagnosis were classified as high risk. Ashkenazi-Jewish women having any first-degree or second-degree relative diagnosed with breast cancer or ovarian cancer were also classified as high risk, except for 1 elevated-risk scenario: having 1 second-degree relative diagnosed with breast cancer at age >50.

### Definition of responder status, characterizing survey salience

We based the threshold for responder status (early vs late) on the 95th percentile of the number of days it took to obtain completed interviews: 30 days for the baseline survey and 27 days for the follow-up survey. For consistency, we used the day-27 threshold for both surveys, where early responders completed interviews by day 27 of the respective survey wave administration, and late responders completed interviews on or after day 28. The maximum number of days required to obtain completed interviews ranged from 31 to 45 days among the baseline waves and from 34 to 44 days among the follow-up waves. Survey salience is indicated for women who have experience relevant to the study research aims. We identified the following baseline variables as salient to survey respondents: objective risk for developing ovarian cancer, personal history of breast cancer, perceived risk of developing ovarian cancer, extent of worry about getting ovarian cancer, number of relatives or friends with cancer, and referral for genetic counseling. 

### Statistical analyses of late responders

We restricted the baseline analysis to waves 2 through 5 (n = 1,711) because wave 1 interviewing was temporarily suspended to correct CATI survey administration programming errors, and wave 6 had an abbreviated 19-day interviewing period in which no respondents met the definition of a late responder. We restricted the follow-up survey analysis to waves 1 through 5 (n = 1,824) because wave 6 had an abbreviated 16-day interview period, precluding any late responders. To measure the likelihood of study participation, we calculated response and refusal rates by objective risk group using unweighted data (17) and conducted statistical testing of both response and refusal rate differences. We conducted a paired analysis of responder status between the baseline and follow-up surveys to determine whether baseline responder status predicted follow-up responder status.

For both the baseline and follow-up surveys, we assessed possible associations between responder status and sociodemographic characteristics (age, race, marital status, education, and annual household income) and between responder status and salient variables. All prevalence estimates were weighted to adjust for differential selection probabilities and nonresponse, while standard errors took into account the stratified study design. We performed the analyses using SUDAAN release 10.0.1 (Research Triangle Institute, Research Triangle Park, North Carolina), setting significance at *P* ≤ .05.

## Results

We identified 152 (8.9%) late and 1,559 early baseline survey responders. Obtaining a completed interview required a median of 30 days among late responders and 11 days among early responders. Women at high or elevated objective risk for ovarian cancer had a 73.4% (988/1,346) response rate, significantly higher (*P* = .04) than the 69.6% (723/1,039) response rate of women at average risk. Conversely, women at high or elevated risk were significantly less likely (*P* = .02) to refuse baseline survey participation (11.1% [149/1,346]) than women at average risk (14.3% [149/1,039]).

We found no significant differences between early and late responders in sociodemographic characteristics (Table 1). Among early responders, 5.2% (95% confidence interval [CI], 4.8%–5.6%) had a personal history of breast cancer compared with 3.4% (95% CI, 2.3%–5.0%) of late responders (*P* = .04), whereas 4.6% (95% CI, 3.5%–6.0%) of early responders had been referred for genetic counseling and only 2.0% (95% CI, 1.3%–3.2%) of late responders had been referred (*P* = .004) (Table 2).

**Table 1:** Baseline Survey Sociodemographics of Overall Study Population and by Responder Status,^a^ Women, Aged 30 or Older, Henry Ford Health System, 2008^b^

Characteristic	Overall Study Population (n = 2,524)		Responder Status^a^ (n = 1,711)	
			Early (n = 1,559)	Late (n = 152)

	n	% (95% CI)	% (95% CI)	% (95% CI)
**Age, y**				
30–39	227	11.7 (10.1–13.3)	10.3 (8.3–12.8)	13.6 (7.1–24.5)
40–49	542	23.7 (21.6–25.7)	26.1 (23.0–29.5)	19.9 (12.2–30.6)
50–59	837	31.5 (29.3–33.8)	32.4 (29.1–35.8)	32.0 (22.4–43.4)
60–64	401	14.0 (12.4–15.6)	12.3 (10.2–14.8)	22.5 (14.1–33.9)
≥65	517	19.1 (17.3–21.0)	18.9 (16.3–21.8)	12.1 (6.8–20.6)
**Race**				
White	1,682	65.2 (62.9–67.5)	62.4 (58.8–65.9)	63.2 (51.3–73.6)
Black	693	28.5 (26.4–30.7)	29.7 (26.5–33.1)	30.9 (21.1–42.7)
American Indian/Alaska Native	22	0.9 (0.4–1.4)	^—^ c	^—^ c
Asian/Native Hawaiian/Other Pacific Islander	42	2.0 (1.3–2.7)	2.6 (1.6–4.1)	^—^ c
Multiracial	44	1.6 (1.0–2.2)	2.1 (1.3–3.5)	^—^ c
Unknown/refused to answer	41	1.7 (1.1–2.3)	2.0 (1.1–3.4)	^—^ c
**Marital status**				
Married/partnered	1,692	67.5 (65.2–69.7)	68.2 (64.7–71.5)	67.2 (55.4–77.2)
Separated/divorced	380	13.7 (12.1–15.3)	13.0 (10.8–15.6)	17.3 (10.0–28.2)
Never married	231	10.7 (9.1–12.2)	11.2 (9.0–13.8)	^—^ c
Widowed	219	8.2 (6.9–9.5)	7.6 (5.9–9.7)	^—^ c
**Education**				
<High school/high school graduate/GED	776	28.9 (26.7–31.0)	28.3 (25.2–31.7)	28.6 (19.4–39.9)
College, <4 y	830	32.5 (30.3–34.7)	33.1 (29.8–36.6)	32.1 (22.4–43.5)
College, ≥4 y	484	20.3 (18.4–22.3)	21.5 (18.7–24.7)	16.8 (10.1–26.7)
Graduate degree	433	18.3 (16.4–20.2)	17.0 (14.5–19.9)	22.6 (14.0–34.2)
**Annual household income** d, **$**				
<25,000	263	10.2 (8.8–11.7)	9.7 (7.7–12.0)	6.6 (2.9–14.6)
25,000 to <50,000	706	26.5 (24.4–28.6)	26.6 (23.5–29.9)	30.3 (20.8–41.8)
50,000 to <75,000	592	23.2 (21.2–25.3)	23.1 (20.2–26.3)	17.8 (10.8–28.1)
≥75,000	962	40.0 (37.7–42.4)	40.7 (37.1–44.3)	45.2 (34.2–56.8)

Abbreviations: CI, confidence interval; GED, general educational development (high school equivalency).

a Early responders completed a survey wave interview within the first 27 days of interviewing; late responders completed interviews on or after day 28. The responder status analysis was restricted to waves 2 through 5 from among 6 total survey waves.

b Percentages do not always sum to 100% because of rounding.

c Suppressed estimates are unstable with relative standard errors >30%, or no sample was observed.

d We used hot-deck imputation procedures to assign the income group for 7.8% (198) of all respondents.

**Table 2:** **Baseline Survey Key Study Variables by** **Responder Status,** a **Women, Aged 30 or Older, Henry Ford Health System, 2008^b^**

Variable	Responder Status^a^ (n = 1,711)				Test of Association *P* value
	Early (n = 1,559)		Late (n = 152)		
	n	% (95% CI)	n	% (95% CI)	
**Objective risk**					
High risk, personal history of breast cancer and family history of breast or ovarian cancer, or personal history of breast cancer only	148	2.2 (2.0–2.5)	9	1.3 (0.8–2.3)	.12
High risk, family history cancer only	340	5.1 (4.8–5.4)	40	6.0 (4.4–8.1)	
Elevated	412	7.1 (6.9–7.3)	39	6.7 (4.9–9.1)	
Average with family cancer history	315	19.3 (18.7–19.9)	28	17.0 (11.9–23.9)	
Average with no family cancer history	344	66.3 (65.4–67.1)	36	68.9 (60.7–76.2)	
**Personal history of breast cancer**					
Yes	321	5.2 (4.8–5.6)	21	3.4 (2.3–5.0)	.04
No	1,238	94.8 (94.4–95.2)	131	96.6 (95.0–97.7)	
**Perceived risk composite score^c^**					
11–15	445	21.5 (18.8–24.5)	53	17.3 (11.2–25.7)	.66
9–10	446	28.8 (25.6–32.2)	41	27.1 (18.1–38.5)	
7–8	313	26.5 (23.3–30.0)	26	26.4 (17.0–38.5)	
4–6	249	23.2 (20.1–26.7)	24	29.3 (19.3–41.7)	
**Worry scale composite score^d^**					
7–11	114	6.3 (4.8– 8.2)	14	^ —^ e	.68
4–6	436	25.3 (22.4–28.6)	38	21.0 (13.3–31.6)	
3	1,009	68.4 (65.0–71.6)	100	72.3 (61.2–81.2)	
**No. of relatives or friends with cancer**					
0 or 1	251	28.0 (24.7–31.5)	29	31.5 (21.5–43.6)	.52
2	251	19.6 (16.8–22.6)	20	15.1 (8.6–25.2)	
3	249	14.5 (12.2–17.2)	32	19.6 (12.1–30.3)	
≥4	808	37.9 (34.6–41.3)	71	33.7 (24.1–44.9)	
**Referred for genetic counseling^f^**					
Yes	156	4.6 (3.5–6.0)	13	2.0 (1.3–3.2)	.004
No	1,398	95.4 (94.0–96.5)	139	98.0 (96.8–98.7)	

a Early responders completed a follow-up survey interview by day 27 of wave interviewing; late responders completed a follow-up survey interview on day 28 or later of wave interviewing.

b Percentages do not always sum to 100% because of rounding.

c The perceived risk composite score is based on 3 questions about the respondents’ perceptions of their risk for developing ovarian cancer. These queries involved perceived risk of developing ovarian cancer in the next 10 years compared with most women their age, perceived lifetime risk, and the influence of family medical history on their personal risk for developing ovarian cancer. The scale ranges from 3 (lowest perceived risk) to 15 (highest perceived risk); reported levels ranged from 4 to 15.

d The worry scale composite score is based on 3 questions that determine the extent to which respondents worried during the past month about getting ovarian cancer. These queries included how often they thought about their chances of developing ovarian cancer, how often such thoughts affected their mood, and how often such thoughts affected their ability to perform their daily activities. The scale ranges from 3 (lowest level of worry) to 15 (highest level of worry); reported levels of worry ranged from 3 to 11.

e The suppressed prevalence estimate is unstable with a relative standard error >30%.

f The baseline survey asked about genetic counseling referral as follows: “Genetic counseling involves a discussion with a health care professional about your family’s history of cancer. Have you ever been referred by a doctor or another health care professional for genetic counseling for cancer risk?”

We identified 90 (4.9%) late responders and 1,734 early responders in the follow-up survey. Obtaining completed interviews required a median of 30 days among late responders and 5 days among early responders. The overall response rate for the follow up was 77.9% (1,824/2,342), whereas the overall refusal rate was only 2.9% (67/2,342). We found no statistically significant differences in either the response rates (range, 77.0%–78.3%) or refusal rates (range, 2.4%–3.4%) across objective risk categories or in sociodemographic characteristics between early and late responders in the follow-up survey. However, consistent with the baseline survey, the prevalence of a personal history of breast cancer in the follow-up was significantly greater (*P* = .01) among early responders 5.7% (95% CI, 5.2%–6.2%) than late responders 3.0% (95% CI, 1.7%–5.2%). Because the follow-up survey did not ask about genetic counseling referral, we could not determine whether referral differences persisted by responder status.

The baseline survey/follow-up survey matched paired observations (n = 1,303 pairs) indicated that baseline survey responder status predicted follow-up survey responder status (*P* = .005), a result driven by the 94.0% (1,119) of baseline early responders (1,190) who were also follow-up early responders. In contrast, baseline survey late responders did not tend to repeat as follow-up survey late responders; 96.5% (109) of late responders at baseline (113) were early responders at follow-up. Nonresponse to the follow-up survey, including noncontact and refusals, did not vary by baseline survey responder status (*P* = .68).

## Discussion

In our study, a personal history of breast cancer and genetic counseling referral were more prevalent among early responders, indicating that women with experience relevant to the study research aims were more likely to respond to the baseline survey. Women at higher risk levels had higher response rates and lower refusal rates than women at average risk. Women with a personal history of breast cancer remained early responders in the follow-up survey. These findings correspond to the importance of survey salience in leveraging women’s participation.

We found no differences in sociodemographic characteristics by responder status in either the baseline or follow-up, as was reported in a survey of pharmacists (7). Although age and sex did not predict response to a survey conducted in Switzerland, lower levels of education and income and a report of poorer health predicted the need for increased reminder mailings (8). Despite a relatively high response rate, a study of health-related behaviors in the Baltic countries found late response to be weakly related to age and education (3). Thus, significant sociodemographic differences by response status indicate that demographic predictors of nonresponse are not uniform across studies (8). We would expect to find differences in demographic characteristics by response status across studies that also differ in target audiences, questionnaire content, duration of survey, and mode of administration.

We found that late responder status was generally discordant between baseline and follow-up, a potentially important finding for survey researchers making recruitment decisions on follow-up of nonresponders to longitudinal surveys. This finding indicates that additional efforts to recruit late responders to a baseline survey will not necessarily translate into similarly prolonged recruitment in follow-up surveys. That our follow-up survey refusal rates did not vary across objective risk categories suggests that perhaps respondents who had already participated in the baseline survey felt equally committed to participating in the follow up, regardless of their medical history. Compliance, or the willingness to cooperate with a request, also has been described as “commitment and consistency,” a heuristic or principle that describes the tendency to behave in a similar way in similar situations (18).

Our introductory survey materials specifically referenced our research interest in women with cancer experience, both personal and in their families. Women who themselves have experienced cancer or who have a significant family history of cancer might be most interested in a survey on “what women know and think about cancer” and “experience of cancer” because they might want an opportunity to describe their own experiences or aid cancer research. We described one of the research goals as facilitating communication between patients and physicians about cancer screening. Thus, the survey might have appeared particularly salient to women who have or have had breast cancer themselves and women who had been referred for genetic counseling. Such women are more likely to have explored their own family cancer histories, either informally or as part of the genetic counseling and BRCA1/2 testing process. In this regard, they might have felt more comfortable responding to a survey focused on family history of cancer. The same characteristics that motivate women to pursue genetic counseling and BRCA1/2 testing might also influence their likelihood to participate in related research.

This study had several strengths. First, we conducted the baseline and follow-up surveys in a large, racially diverse population that included nearly 30% African-American women, an understudied group with regard to hereditary breast and ovarian cancer. We also recruited a large sample of women at high, elevated, and average risk from a general population setting. Furthermore, our large sample facilitated a detailed exploration of the characteristics of early versus late responders in the baseline survey and a comparison of response timing between the baseline and follow-up surveys. Finally, we achieved a very good baseline survey response rate (76.3%) and retained 75.7% of the baseline respondents in the follow-up to conduct a longitudinal assessment of response propensity and timing.

One study limitation was the self-report of family cancer history. Studies have found reporting of first-degree relatives to be more accurate than that of second-degree relatives (19–21) and reporting of breast cancer to be more accurate than reporting of ovarian cancer (20,21). Thus, our objective risk classification might have underestimated the number of women at high risk, thereby attenuating differences among risk groups. Another limitation is related to the restriction of our data collection to 1 health system in the Midwest. Thus, the results might not be generalizable to other regions of the United States where attitudes about health, cancer, and personal openness might differ.

Another concern is the potential difference in data quality between early and later responders. To address these data quality concerns, we assessed the extent of missing data among demographic variables, which are asked at the end of the baseline survey, and among other salient study variables, including “don’t know” and refused responses. We found no differences between early and later responders in either the baseline or follow-up surveys. These findings provide evidence of sustained survey data quality among late responders. Our study also found no difference between early and late responders in education level, which might influence data quality. One study has shown that respondents who required a great deal of effort to attain were comparable to early responders (22). However, as we found differences in salient study variables by responder status, the pursuit of late responders in our study might have reduced response bias, at least with respect to salient study variables.

Because we do not know the exact dates of the first call attempts to responders in either the baseline or follow-up surveys, we assumed that all responders were initially contacted on the first interview day of their survey wave. Because initial call attempts occurred within the first 8 to 10 days of baseline survey wave administration and within the first 3 to 8 days of follow-up survey wave administration, we potentially misclassified some early responders as late responders. Consequently, we consider observed differences by responder status to be conservative.

The introduction of a survey topic at the critical first solicitation, either through letters or telephone scripts, is important in study recruitment. Using only generic introductory language to state survey purpose, such as to understand health in a general way, might not garner the desired response. Our findings suggest that participants are more likely to respond if the survey is of interest to them or if they believe that their responses will be deemed important. This is perhaps more relevant for special surveys than general health surveys. In addition to salience, our favorable response rate was likely bolstered by other elements shown to enhance recruitment: incentives for baseline and follow-up surveys and acknowledgment of respondents’ time and effort through thank-you letters. Finally, research also suggests that sponsorship by a legitimate authority enhances response (2).

To our knowledge, no published research has assessed longitudinal survey response patterns or potential response bias between early and later respondents in a survey involving self-reports. Our findings on establishing salience in introductory survey materials should help inform survey research and cancer or chronic disease prevention research in particular. Finally, we suggest that the extra effort made in recruiting late responders is a wise investment in time and resources.
